# The Serum Concentrations of Hedgehog-Interacting Protein, a Novel Biomarker, Were Decreased in Overweight or Obese Subjects

**DOI:** 10.3390/jcm10040742

**Published:** 2021-02-12

**Authors:** Hsuan-Wen Chou, Hao-Chang Hung, Ching-Han Lin, An-Chi Lin, Ye-Fong Du, Kai-Pi Cheng, Chung-Hao Li, Chih-Jen Chang, Hung-Tsung Wu, Horng-Yih Ou

**Affiliations:** 1 Division of Endocrinology and Metabolism, Department of Internal Medicine, National Cheng Kung University Hospital, College of Medicine, National Cheng Kung University, Tainan 70403, Taiwan; coolpikachu2007@gmail.com (H.-W.C.); haochang.hung@gmail.com (H.-C.H.); cyclops0113@yahoo.com.tw (C.-H.L.); fiyonlin@gmail.com (A.-C.L.); jamesdu.du@gmail.com (Y.-F.D.); supercabyhome@yahoo.com.tw (K.-P.C.); 2 Department of Health Management Center, National Cheng Kung University Hospital, National Cheng Kung University, Tainan 70403, Taiwan; smallhear@gmail.com; 3 Department of Family Medicine, National Cheng Kung University Hospital, College of Medicine, National Cheng Kung University, Tainan 70403, Taiwan; changcj.ncku@gmail.com; 4 Graduate Institute of Metabolism and Obesity Sciences, College of Nutrition, Taipei Medical University, Taipei 11031, Taiwan

**Keywords:** hedgehog-interacting protein, impaired fasting glucose, impaired glucose tolerance, newly diagnosed diabetes, normal glucose tolerance

## Abstract

Although it was known that obesity is an independent risk factor for metabolic disorders including diabetes, the factors that link these diseases were obscure. The Hedgehog-interacting protein (Hhip) is a negative regulator in tissue remodeling, and inhibits the proliferation of adipocytes, and promotes their differentiation. In addition, Hhip was positively associated with diabetes. However, the relationship between Hhip and obesity in the human body remains unclear. An analysis of the relationship between Hhip and normal weight, overweight, and obesity levels. Participants receiving a physical checkup were recruited. Anthropometric and biochemical data were collected. Serum Hhip levels were determined by enzyme-linked immunosorbent assay (ELISA). Subjects were classified into normal-weight, overweight, and obese groups based on their body mass index (BMI). The association between Hhip and obesity was examined by multivariate linear regression analysis. In total, 294 subjects who were either of a normal weight (*n* = 166), overweight (*n* = 90), or obese (*n* = 38) were enrolled. Hhip concentrations were 6.51 ± 4.86 ng/mL, 5.79 ± 4.33 ng/mL, and 3.97 ± 3.4 ng/mL in normal-weight, overweight, and obese groups, respectively (*p* for trend = 0.032). Moreover, the regression analysis showed that BMI (β = −0.144, 95% confidence interval (CI) = −0.397−0.046, *p =* 0.013) was negatively associated with Hhip concentrations after adjusting for sex and age. Being overweight (β = −0.181, 95% CI = −3.311−0.400, *p =* 0.013) and obese (β = −0.311, 95% CI = −6.393−2.384, *p* < 0.001) were independently associated with Hhip concentrations after adjusting for sex, age, fasting plasma glucose, the insulin level, and other cardiometabolic risk factors. Our results showed that overweight and obese subjects had lower Hhip concentrations than those of normal weight. Being overweight and obese were negatively associated with Hhip concentrations. Hhip might be a link between obesity and diabetes.

## 1. Introduction

Obesity is recognized as an independent risk factor for the development of many diseases, such as diabetes mellitus, cardiovascular diseases, and even cancer [1,2,3]. The World Health Organization defines obesity as abnormal or excessive fat accumulation that may impair health [4]. In clinical practice, the body mass index (BMI) has been used for diagnosing obesity and being overweight [5]. An energy imbalance of more calories being consumed than expended is the most important cause of obesity, and the consequence is the storage of excess energy in adipose tissues that increase in size by hypertrophy and hyperplasia [6,7].

The Hedgehog (Hh) signaling pathway is known to be an important pathway for the growth, development, and homeostasis of many tissues in animals, especially during embryonic development [8]. Recently, Hh signaling was proven to be related to adipose tissue differentiation [9,10,11,12]. Activation of Hh signaling inhibits adipocyte differentiation in vitro [9]. Targeted activation of Hh signaling suppresses high-fat-diet-induced obesity and improves whole-body glucose tolerance and insulin sensitivity in vivo [10]. Because the Hh signaling pathway was reported to be involved in adipogenesis, it was proposed as a potential therapeutic target for metabolic diseases such as type 2 diabetes and obesity [11,13].

The Hh-interacting protein (Hhip), a membrane glycoprotein, is a negative regulator that attenuates Hh signaling by binding to its ligands [14,15]. During 8-day adipocyte differentiation, Hhip messenger RNA and protein expressions peaked at day 6 in 3T3-L1 cells [13]. In addition, Hhip messenger RNA expression in adipose tissues was higher in 3-day-old than in 180-day-old pigs [13]. Recombinant Hhip treatment promoted 3T3-L1 cell differentiation by upregulating the expression of peroxisome proliferator-activated receptor γ and glucose transporter 4 and downregulating the expression of the Hh signaling transcription factor, Gli1 [13]. We previously reported that the Hhip was positively associated with prediabetes and type 2 diabetes [16]. Because obesity is closely associated with dysglycemia [1], we explored the relationship between Hhip levels and being overweight/obese in humans in this study.

## 2. Materials/Subjects and Methods

### 2.1. Participants

This study was approved by the Institutional Review Board of National Cheng Kung University Hospital (ER-104-204) (Tainan, Taiwan), and all participants signed an informed consent form before joining the study. All participants in the study were recruited between January 2016 and December 2016 from the Health Examination Center of National Cheng Kung University Hospital.

Blood was sampled at 9:00 from all participants after they had fasted for 12 h overnight. Subjects without a history of diabetes received an oral glucose tolerance test. After fasting blood sampling, subjects were instructed to drink 75 g glucose in 300 mL water within 5 min. Two hours after drinking glucose solution (11 am), a blood sample was collected again to measure blood glucose level. Those who (1) had an acute or chronic inflammatory disease as determined by a leukocyte count of >10,000/mm^3^ or clinical signs of infection; (2) had any other major diseases, including generalized inflammation or advanced malignant diseases contraindicating this study; (3) were pregnant; (4) had a history of diabetes and were receiving insulin therapy, glucagon like-peptide-1, or oral antidiabetic drugs; (5) were taking drugs that affect glucose homeostasis, such as corticosteroids, thiazides, etc.; (6) had experienced an acute coronary syndrome, cerebrovascular accident, or pancreatitis during the past three months; or (7) were taking lipid-lowering medications or antihypertensive drugs were excluded.

We grouped all participants into one of three groups according to the recommendations of the Health Promotion Administration of Taiwan based on their BMI—normal weight (18.5 kg/m^2^ < BMI < 24 kg/m^2^), overweight (BMI ≥ 24 kg/m^2^), and obese (BMI ≥ 27 kg/m^2^) [17].

### 2.2. Data Collection

We measured every subject’s body height and waist circumference to the nearest 0.1 cm and body weight (BW) to the nearest 0.1 kg. The BMI was defined as the BW (kg) divided by the body height (m) squared. We asked participants to rest in the supine position in a quiet place to measure the blood pressure between 08:00 and 10:00 while in a fasted status. An appropriate-sized cuff was used for the right upper arm, and the pressure was checked twice at an interval of at least 5 min using a DINAMAP vital signs monitor (model 1846SX; Critikon, Irvine, CA, USA). The hexokinase method (Roche Diagnostic, Mannheim, Germany) was used to measure the blood glucose. An enzyme-linked immunosorbent assay (ELISA) (Mercodia AB, Uppsala, Sweden) was used to measure serum insulin levels. A highly sensitive ELISA kit (Immunology Consultants Laboratory, Newberg, OR, USA) was used to determine high-sensitivity C-reactive protein. A human Hhip ELISA kit (MyBioSource, San Diego, CA, USA) was used for determining serum Hhip concentrations. The intra-assay coefficient of variation of the ELISA was 5.52% and the inter-assay coefficient of variation of it was 4.9%. An autoanalyzer (Hitachi 747E; Tokyo, Japan) in the central laboratory of National Cheng Kung University was employed to obtain serum alanine aminotransferase, aspartate aminotransferase, total cholesterol, triglycerides, high-density lipoprotein cholesterol, and low-density lipoprotein cholesterol. A high-performance liquid chromatographic method (Tosoh Automated Glycohemoglobin Analyzer; Tokyo, Japan) was used to measure glycated hemoglobin (HbA1c). The estimated glomerular filtration rate (eGFR) was calculated by the modification of the diet in a renal disease equation. The homeostasis model assessment of insulin resistance was defined by the formula that is equal to fasting insulin (mU/L) multiplied by fasting plasma glucose (mg/dl) divided by 405 to investigate insulin resistance [18].

### 2.3. Statistical Analyses

Data were analyzed using SPSS software (vers. 24.0; SPSS, Chicago, IL, USA). Baseline characteristics are expressed as the mean ± standard deviation (SD) for continuous variables or as a percentage for categorical variables. A one-way analysis of variance (ANOVA) was used to determine any difference in variables among the groups. Chi-square tests were used to analyze differences in categorical variables among the groups. The Bonferroni correction was used for a post hoc study to see if serum Hhip concentrations differed among the groups. A multivariate linear regression analysis was performed to identify independent variables related to serum Hhip concentrations. The criterion for statistical significance was a *p*-value of <0.05.

## 3. Results

**Figure 1:**
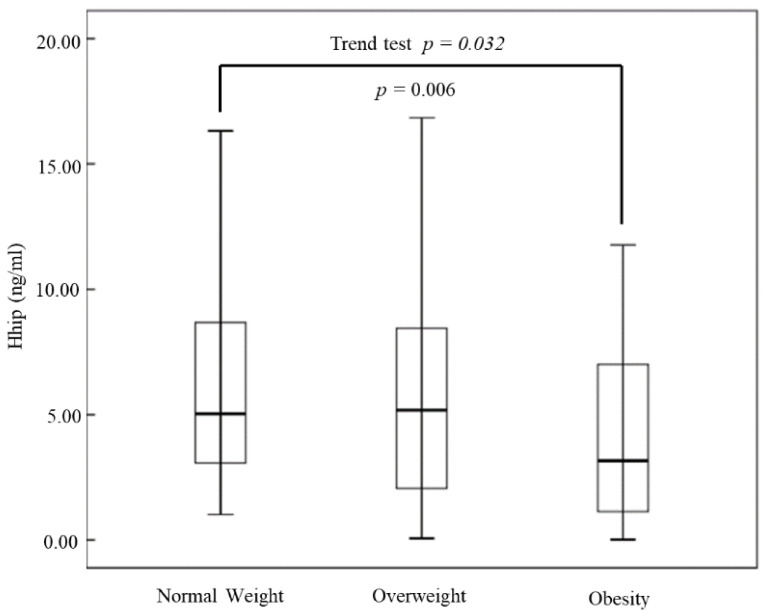
Comparisons of serum concentrations of the Hedgehog-interacting protein (Hhip) in normal-weight, overweight, and obese subjects. Box and whisker plot of serum Hhip concentrations in participants with normal-weight (*n* = 166), overweight (*n* = 90), and obese subjects (*n* = 38). The line inside the box represents the median of the distribution, the box top, and bottom values are defined by the 25th and 75th percentiles, and the whiskers are minimum and maximum values.

Overall, 294 subjects were enrolled and classified into the normal-weight (*n* = 166), overweight (*n* = 90), and obese (*n* = 38) groups. The average age of them was 61.34 ± 11.87-year-old. Comparisons of baseline characteristics of these participants are shown in Table 1. There were significant differences in the BW (*p* < 0.001), waist circumference (*p* < 0.001), BMI (*p* < 0.001), diastolic blood pressure (*p* = 0.022), HbA1c (*p* = 0.026), high-density lipoprotein cholesterol (*p* = 0.008), triglycerides (*p* = 0.008), homeostasis model assessment of insulin resistance (*p* < 0.001), and insulin levels (*p* < 0.001) among the three groups. Hhip concentrations were 6.51 ± 4.86 ng/mL, 5.79 ± 4.33 ng/mL, and 3.97 ± 3.4 ng/mL in the normal-weight, overweight, and obese groups, respectively (Figure 1, trend test *p* = 0.032). In the post hoc analysis, serum Hhip concentrations were significantly lower in the obese group (*p* = 0.006), compared to the normal-weight group. Serum Hhip concentrations were not different between the overweight group and the normal-weight group (*p* = 0.667). Moreover, after exclusion of the study subjects with diabetes, the serum Hhip concentrations were 5.99 ± 4.86 ng/mL, 5.28 ± 3.91 ng/mL, and 4.1 ± 3.55 ng/mL in the normal-weight, overweight, and obese groups. We still observed that serum Hhip concentrations significantly decreased among groups using post hoc analysis (*p* for trend test was 0.044).

In the multivariate linear regression analysis (Table 2), the BMI (β = −0.144, 95% CI = −0.397−0.046, *p* = 0.013) was negatively associated with Hhip concentrations after adjusting for sex and age (model 1). After adding the fasting glucose and insulin levels as confounding factors into model 1, the BMI (β = −0.147, 95% CI = −0.411−0.041, *p* = 0.017) was still independently associated with Hhip concentrations (model 2). To evaluate if the BMI status made a difference in Hhip concentrations, the normal-weight group was used as a reference to compare with the overweight and obese groups. We found that being overweight (β = −0.181, 95% CI = −3.311−0.400, *p* = 0.013) and obese (β = −0.311, 95% CI = −6.393~−2.384, *p* < 0.001) were independently negatively associated with Hhip concentrations after adjusting for sex, age, fasting plasma glucose, insulin level, high-sensitivity C-reactive protein, systolic blood pressure, the estimated glomeruli filtration rate (eGFR), alanine aminotransferase, cholesterol, triglycerides, high-density lipoprotein cholesterol, and low-density lipoprotein cholesterol (model 3).

## 4. Discussion

To the best of our knowledge, this is the first human study to explore the relationship between obesity and the Hhip. We found that Hhip levels progressively decreased from the normal-weight and overweight groups to the obese group. In addition, the BMI was negatively associated with serum Hhip concentrations. Moreover, being overweight and obese were negatively associated with serum Hhip concentrations.

According to our previous study, the presence of prediabetes and type 2 diabetes was positively associated with serum Hhip concentrations, while the BMI was not [16]. However, the average BMI in the previous study was similar among subjects with normal glucose tolerance (BMI = 22.3 kg/m^2^), impaired fasting glucose (BMI = 23.6 kg/m^2^), impaired glucose tolerance (BMI = 23.4 kg/m^2^), and newly diagnosed diabetes (BMI = 23.3 kg/m^2^), although the difference reached borderline statistical significance (*p* = 0.049), which may be at risk of a type 1 error, and subjects with obesity might not have been included. It is therefore unknown whether or not being overweight/obese is associated with plasma Hhip concentrations. Wei et al. reported that recombinant Hhip can increase adipocyte differentiation, which results in increased accumulation of lipid droplets in adipocytes by inhibiting the Hh signaling pathway in 3T3-L1 cells, and Hhip messenger RNA expression in adipose tissues was lower in 180-day-old than in 3-day-old pigs [13]. It was suggested that serum Hhip concentrations may be negatively regulated by differentiated adipose tissues. Once one becomes obese, the production of Hhip should decrease to prevent further adipocyte differentiation. However, the mechanism as to how adipose tissues influence serum Hhip concentrations remains unclear. To address this hypothesis, further human studies are required. In our cohort, obese subjects had a lower level of Hhip protein, which can be viewed as a mechanism of negative feedback, in order to promote the Hh pathway and inhibit fat formation. We still found that serum Hhip concentrations significantly decreased in the obese group after excluding subjects with diabetes. Moreover, in our previous study, subjects with prediabetes or type 2 diabetes have a higher level of Hhip concentrations. We, therefore, speculated that elevated Hhip concentrations might be a hint that these overweight or obese subjects may have a risk to progress into diabetes compared with those who have lower Hhip concentrations. Hh signaling plays an important role in inhibiting fat formation [11]. A previous animal study showed the activation of Hh signaling decreased obesity induced by a high-fat diet in adult mice [10], and a deficiency of Hh signaling in myeloid cells increased the BW of mice [19]. In obese subjects, the circulating leptin level increases. Wang et al. reported that leptin decreased the weight of obese mice induced by a high-fat diet and inhibited Gli1 expression [20]. In a human study, expression of the Hh signaling transcription factor, Gli1, significantly decreased in adipose tissues of insulin-sensitive obese subjects compared to lean subjects, which may indicate that Hh signaling decreases in obese humans [21]. Circulating Hh ligands and expressions of Hh ligands in adipose tissues increased in obese mice. However, serum Hh ligand levels significantly decreased in morbidly obese (BMI > 40 kg/m^2^) people, even in those with HbA1c > 7%, possibly due to the inhibitory effect of metformin on Hh ligand expression in adipose tissues [19]. As leptin negatively regulates Hh signaling by decreasing Gli1 expression and Hhip is a negative regulator that attenuates Hh signaling by binding to Hh ligands, we may speculate that decreased serum Hhip concentrations in obese subjects are a compensatory mechanism of decreasing Gli1 expression. However, further study is needed to clarify the regulatory architecture. Cholesterol has been shown to be an endogenous Smoothened activator, which is a second messenger activating the Hedgehog signaling pathway [22]. Exogenously added cholesterol would activate Hh signaling pathway in vitro [23]. Cholesterol is not just necessary but also sufficient to activate signaling by the Hh pathway [24]. Hh signaling plays an important role in inhibiting fat formation [11], which means that elevated cholesterol levels might activate Hh signaling to minimize fat formation at the same time that Hhip should be downregulated to avoid fat formation. However, there has been no human study to discuss the relationship between cholesterol and Hhip so far. In our study, we found no difference in cholesterol levels among the three groups. Moreover, in multivariate linear regression analysis, cholesterol was not an independent factor of serum Hhip concentrations. The relationship among cholesterol level, Hh signaling pathway, and serum Hhip concentrations needs to be evaluated in a human study. There were some limitations in this study. First, this study was designed as a cross-sectional study which did not allow for causal inferences between serum Hhip concentrations and BMI or obesity. Second, although one study revealed that the Hhip was associated with moderate to severe chronic obstructive pulmonary disease, all of our participants were apparently healthy with no airway symptoms [25]. Third, we could not directly measure Hhip expression by adipose tissues. Therefore, we could not be sure whether serum Hhip concentrations were representative of those in adipose tissues. Finally, all study subjects were Taiwanese, and thus our findings might not be applicable to other ethnicities.

## 5. Conclusions

Our results demonstrated that serum Hhip concentrations were negatively associated with BMI, and obese subjects had lower serum Hhip concentrations than normal-weight subjects. Further research is needed to explore the pathophysiological roles and clinical implications of the Hhip in obesity.

## Figures and Tables

**Table 1:** Comparisons of clinical parameters among normal-weight, overweight, and obese subjects.

Clinical Parameters	Normal Weight	Overweight	Obese	*p*
*n*	166	90	38	
Female (%)	42.8	31.1	44.7	0.147
Hypertension (%)	17.1	23.3	27	0.272
Diabetes (%)	24.7	31.1	31.6	0.459
Age (years)	61.7 ± 11.9	60.5 ± 11.8	61.4 ± 12.3	0.736
Body weight (kg)	56.4 ± 7	67.59 ± 7.47	75.79 ± 10.8	<0.001
Waist circumference (cm)	78.37 ± 6.82	87.51 ± 5.77	94.43 ± 8.44	<0.001
Body-mass index (kg/m^2^)	21.65 ± 1.87	25.44 ± 0.88	28.49 ± 1.75	<0.001
SBP (mmHg)	125.3 ± 18.1	128.5 ± 16.1	131.5 ± 16.7	0.098
DBP (mmHg)	72.2 ± 10.4	74.9 ± 9.7	76.8 ± 12.0	0.022
FPG (mg/dL)	101.78 ± 43.28	107.36 ± 35.33	116.47 ± 49.68	0.131
Post-load 2-h glucose (mg/dL)	155.34 ± 80.21	147.54 ± 68.90	175.41 ± 97.48	0.234
HbA1c (%)	6.13 ± 1.28	6.24 ± 1.05	6.79 ± 2.09	0.026
ALT (U/L)	25.51 ± 19.50	35.19 ± 53.50	24.89 ± 10.43	0.068
AST (U/L)	27.23 ± 13.70	30.56 ± 41.84	24.68 ± 25.45	0.429
Creatinine (mg/dL)	0.87 ± 0.20	0.87 ± 0.18	0.88 ± 0.20	0.886
eGFR	90.63 ± 19.76	91.72 ± 15.65	88.22 ± 19.24	0.62
hsCRP (mg/L)	3.68 ± 7.52	3.73 ± 6.27	4.48 ± 4.71	0.809
HDL-C (mg/dL)	55.73 ± 15.39	49.98 ± 13.55	51.74 ± 12.49	0.008
LDL-C (mg/dL)	126.33 ± 33.89	129.02 ± 35.91	131.28 ± 44.65	0.694
Triglycerides (mg/dL)	110.47 ± 68.96	127.56 ± 60.71	144.94 ± 70.84	0.008
Triglycerides (mg/dL) *	1.99 ± 0.21	2.06 ± 0.20	2.12 ± 0.20	<0.001
Cholesterol (mg/dL)	204.16 ± 39.555	204.51 ± 41.545	212.00 ± 47.594	0.56
HOMA-IR	0.50 ± 0.47	0.97 ± 1.50	1.26 ± 1.011	<0.001

Data are expressed as the mean ± standard deviation or as a percentage. * Values were log-transformed before analysis. Hhip, Hedgehog-interacting protein; SBP, systolic blood pressure; DBP, diastolic blood pressure; FPG, fasting plasma glucose; HbA1c, glycated hemoglobin; ALT, alanine aminotransferase; AST, aspartate aminotransferase; eGFR, estimated glomeruli filtration rate; hsCRP, high-sensitivity C-reactive protein; HDL-C, high-density lipoprotein cholesterol; LDL-C, low-density lipoprotein cholesterol; HOMA-IR, homeostasis model assessment of insulin resistance.

**Table 2:** Results of multivariate linear regression analysis between the Hedgehog-interacting protein (Hhip) and clinical variables.

Variable	Model 1		Model 2		Model 3	
	β (95% CI)	*p*	β (95% CI)	*p*	β (95% CI)	*p*
Age (years)	−0.029 (−0.056~0.033)	0.612	−0.034 (−0.058~0.032)	0.565	−0.006 (−0.062~0.057)	0.938
Sex	0.143 (0.278~2.412)	0.014	0.134 (0.182~2.350)	0.022	0.158 (0.204~2.857)	0.024
Body-mass index	−0.144 (−0.397~−0.046)	0.013	−0.147 (−0.411~−0.041)	0.017		
OW vs. NW					−0.181 (−3.311~−0.400)	0.013
OB vs. NW					−0.311 (−6.393~−2.384)	<0.001
Fasting glucose (mg/dL)			0.024 (−0.010~0.015)	0.694	0.007 (−0.015~0.016)	0.925
Insulin (mIU/L)			−0.011 (−0.210~0.175)	0.859	0.049 (−0.175~0.354)	0.503
hsCRP (mg/L)					0.016 (−0.078~0.100)	0.809
SBP (mmHg)					0.06 (−0.021~0.054)	0.397
eGFR					−0.026 (−0.042~0.029)	0.715
ALT (U/L)					−0.07 (−0.027~0.008)	0.296
CHOL (mg/dL)					−0.063 (−0.147~0.133)	0.923
TGs (mg/dL) *					0.029 (−8.388~9.701)	0.886
HDL-C (mg/dL)					−0.101 (−0.182~0.116)	0.663
LDL-C (mg/dL)					0.129 (−0.126~0.158)	0.821

* Values were log-transformed before analysis. OW, overweight; NW, normal weight; OB, obese; hsCRP, high-sensitivity C-reactive protein; SBP, systolic blood pressure; ALT, alanine aminotransferase; eGFR, estimated glomerular filtration rate; CHOL, cholesterol; TGs, triglycerides; HDL-C, high-density lipoprotein cholesterol; LDL-C, low-density lipoprotein cholesterol.

## Data Availability

Part of the data generated in this study is included in the article. Patient and clinicopathological data in this cannot be made publicly available due to their content of identifiable human data. Requests to access the datasets should be directed to Horng-Yih Ou.

## Abbreviations

BMI	body mass index
ALT	alanine aminotransferase
AST	aspartate aminotransferase
DBP	diastolic blood pressure
eGFR	estimated glomeruli filtration rate
FPG	fasting plasma glucose
HbA1c	glycated hemoglobin
HDL-C	high-density lipoprotein cholesterol
Hhip	Hedgehog-interacting protein
HOMA-IR	homeostasis model assessment of insulin resistance
hsCRP	high-sensitivity C-reactive protein
LDL-C	low-density lipoprotein cholesterol
SBP	systolic blood pressure
